# Antioxidant Activities and Prebiotic Activities of Water-Soluble, Alkali-Soluble Polysaccharides Extracted from the Fruiting Bodies of the Fungus *Hericium erinaceus*

**DOI:** 10.3390/polym15204165

**Published:** 2023-10-20

**Authors:** Haining Zhuang, Huayue Dong, Xiaowei Zhang, Tao Feng

**Affiliations:** 1 School of Food and Tourism, Shanghai Urban Construction Vocational College, Shanghai 201415, China; witwheat@163.com; 2 School of Perfume and Aroma Technology, Shanghai Institute of Technology, Shanghai 201418, China; huayuedong2022@163.com; 3 School of Medical Instrument and Food Engineering, University of Shanghai for Science and Technology, Shanghai 200093, China; zhangx@usst.edu.cn

**Keywords:** *Hericium erinaceus* polysaccharides, extraction method, antioxidant, in vitro fermentation, gut microbiome

## Abstract

In this study, the digestion and fermentation properties of the bioactive water-soluble polysaccharide (HEP-W), and alkali-soluble polysaccharide (HEP-A) from *Hericium erinaceus* and the impact on the human colonic microbiota were determined using simulated saliva–gastrointestinal digestion and human fecal fermentation models in vitro. The basic physicochemical properties of HEP-W and HEP-A were determined at the same time. The results showed that the in vitro simulated digestion had almost no effect on the physicochemical properties of HEP-W and HEP-A, indicating that HEP-W and HEP-A were partially degraded. During fermentation, HEP-W and HEP-A increased the relative abundance of the dominant butyric acid-producing genera, the microbial community structure was significantly regulated, the gas production and short-chain fatty acid production in the fermentation broth were significantly increased, and the pH of the fermentation broth was reduced. There were structural and other differences in HEP-W and HEP-A due to different extraction methods, which resulted in different results. These results suggest that HEP-W and HEP-A may be potential gut microbial manipulators to promote gut health by promoting the production of beneficial metabolites by intestinal microorganisms using different butyric acid production pathways.

## 1. Introduction

The gut microbiota are the set of microorganisms that colonize our digestive tract, including a large number of microbial species. The human intestinal microbiota are dominated by *Firmicutes*, *Bacteroides*, *Proteobacteria*, and *Actinobacteria*, which play an important role in physiology and maintaining host health. Gut microbiota are primarily linked with the degradation of carbohydrates, proteins, lipids, and peptides by fermentation and anaerobic degradation, and the related metabolites are used as energy substances or signal molecules for the host [1]. Gut microbiota make important contributions to host nutrient absorption, including the degradation of nondegradable polysaccharides and oligosaccharides, protein metabolism, amino acid synthesis, and the synthesis of vitamins [2,3,4]. Plant-derived polysaccharides, as an important class of bioactive natural products, have attracted increasing attention due to their multiple biological activities, including antioxidant, anticancer, hypoglycemic, anti-inflammation, and immunological activities [5,6,7]. Polysaccharides depend on the metabolism of gut microbes, so their activity is mainly related to changes in microbes. A previous study reported that after feeding mice different concentrations of selenium-rich edible bacteria, the relative abundance of *bifidobacteria* and lactic acid bacteria in the intestines of mice increased, while the content of *Escherichia coli* decreased [8]. Mitsou [9] selected cyclosporine, lion’s mane, oyster mushroom and flat mushroom cultured in various substrates, fermented and cultured the feces of healthy volunteers over 65 years old, and found that the levels of *bifidobacterial*, *lactobacillus* and *Fusobacterium priralii* in volunteers increased significantly.

Due to its rarity, *Hericium erinaceus* is also commonly known as the “mountain treasure”. In recent years, *Hericium erinaceus* has been shown to contain a variety of active ingredients, including polysaccharides, diterpenes, pyranones, phenols, and sterols [10], which have the functions of antioxidant [11], strengthening the spleen and nourishing the stomach [12], protecting the gastric mucosa [13] and anti-tumor [14]. *Hericium erinaceus* polysaccharides can regulate the ecological balance of human intestinal microorganisms by providing essential nutrients for intestinal microorganisms. At present, the extraction methods of *Hericium erinaceus* polysaccharide mainly include enzyme extraction, microwave extraction [15], ultrasonic extraction [16], hot water extraction [17,18,19], chemical extraction (alkali extraction, acid extraction) [20,21], etc. Due to different preparation methods, the structure, molecular weight, and other properties of the resulting *Hericium erinaceus* polysaccharides can also vary greatly. In terms of solubility, are they mainly water-soluble and alkali-soluble, in terms of molecular weight, their order of magnitude is in the range of 105–106, and in terms of structure, they mainly consist of β-(1→3) and β-(1→6)-glycosidic bonds, in which the molar ratio of the two types of glycosidic bonds differs greatly [22,23,24,25,26]. The significant structural differences in the β-glucan of *Hericium erinaceus* can be used as an ideal representative of the β-glucan of edible bacteria, providing a good model for studying the effect of different preparation methods and different structures on the regulation of the species structure of human fecal intestinal flora.

Not many studies have been conducted to investigate the relationship between different extraction methods of *Hericium erinaceus* polysaccharides and human intestinal microorganisms. Therefore, this study aimed to investigate the digestion and fermentation characteristics of HEP-W and HEP-A using simulated saliva–gastrointestinal digestion and human fecal fermentation models in vitro, by studying the digestion and fermentation characteristics of *Hericium erinaceus* polysaccharides obtained by different extraction methods, and analyzing their basic physicochemical properties and antioxidant activities. The results promise to provide useful information for the application of *Hericium erinaceus* polysaccharide as a prebiotic additive in functional food.

## 2. Materials and Methods

### 2.1. Materials

*Hericium erinaceus* was purchased from the Yongding Ecological Farming Family Farm, Ningqiang County, Hanzhong City, Shaanxi Province.

Saliva α amylase (2000 U/g) was obtained from Omega, Cheyenne, WY, USA. Pepsin (250 U/g), pancreatic enzymes (10 U/g), fructose oligosaccharide, salt acid, concentrated sulfuric acid, sodium hydroxide, sodium chloride, ferrous sulfate heptahydrate, manganese sulfate, zinc sulfate heptahydrate, cobalt chloride hexahydrate, nickel chloride, copper sulfate pentahydrate disodium phosphate, potassium chloride, urea, magnesium chloride hexahydrate, calcium chloride dihydrate, metaphosphate, sodium sulfate, acetic acid standard, propionic acid standard, and butyric acid standard were obtained from Sinopharm Group Chemical Reagent Co., Ltd., Shanghai, China. All other chemical reagents were of analytical grade and procured from Aladdin Chemical Reagent Co., Ltd. (Shanghai, China).

### 2.2. Preparation of Two Hericium erinaceus Polysaccharides

Water-soluble polysaccharide (HEP-W): Dried and ground *Hericium erinaceus* was treated twice with distilled water in the proportion of 1:15 (g /mL) at 100  °C for 2  h. After the *Hericium erinaceus* polysaccharide extraction was completed, filtrate was filtered and collected, and filtrate was concentrated in vacuum in a 1:5 ratio. The concentrate was naturally cooled to room temperature and centrifuged at 8000  rpm for 15  min at 25 °C, and the protein was removed using the Sevage method (polysaccharide extraction: reagents (trichloromethane: n-butanol = 4:1) = 5:1). The polysaccharide solution of protein was removed and centrifuged, and the supernatant was collected and mixed with four volumes of 75% ethanol (*v*/*v*) for 24  h at 4 °C. After alcohol precipitation, the supernatant was removed at 8000  rpm for 15  min at 4 °C, and then distilled water was added under stirring at 50 °C for 1 h to obtain polysaccharide solution. Then, the *Hericium erinaceus* polysaccharide solution was dialyzed (3500 Da) to remove small molecules and inorganic ions. Finally, dialyzed solution of polysaccharide was vacuum dried to obtain polysaccharide powder [25].

Alkali-soluble polysaccharide (HEP-A): Dried and ground *Hericium erinaceus* was treated with 1.25 mol/L NaOH/0.05% NaBH4 in the proportion of 1:10 (g/ mL) at 25  °C for 3  h for two times and then precipitated with 36% acetic acid at pH = 7 (±0.1). After the *Hericium erinaceus* polysaccharide extraction was completed, filtrate was filtered and collected, and filtrate was concentrated in vacuum in a 1:5 ratio. The subsequent operation is similar to the water-soluble polysaccharide to obtain polysaccharide powder [21].

### 2.3. Determination of Sugar Content

#### 2.3.1. Determination of Total Sugar

0.1 mg/mL of HEP-W solution and HEP-A solution was prepared in 10 mL test tubes. The total sugar content of *Hericium erinaceus* polysaccharide was measured using phenol-sulfuric acid method [27].

#### 2.3.2. Determination of Reducing Sugar

1 mg/mL of HEP-W solution and HEP-A solution were prepared in 10 mL test tubes. Reducing sugar content was analyzed using 3, 5-dinitrosalicylic acid method [28].

#### 2.3.3. Determination of Uronic Acid

2 mg/mL of HEP-W solution and HEP-A solution were prepared in 10 mL test tubes, adding sulfuric acid–carbazole reagent to *Hericium erinaceus* polysaccharide solution [29].

### 2.4. Determination of Molecular Weight (Mw)

The Mw of *Hericium erinaceus* polysaccharide was analyzed using high-performance gelfiltration chromatography (HPGFC).

8 mg/mL of HEP-W solution and HEP-A solution were prepared and passed through a 0.22 μm aqueous membrane, then the solution was ready to be measured. Fucose, amino galactose, rhamnose + arabinose, glucosamine, galactose, glucose, xylose, mannose, fruit sugar, galacturonic acid, and glucuronic acid were formulated into a mixed standard solution.

Program settings:

Instrument: Waters (Milford, MA, USA) 1525 high-performance liquid chromatograph (2410 RID and Empower work station);

Chromatographic column: Ultrahydrogel^TM^ Linear 300 mm × 7.8 mm;

Mobile phase: 0.1N NaNO_3_

Flow: 0.5 mL/min

Column temperature: 40 °C.

### 2.5. Determination of Monosaccharide Composition

The monosaccharide composition of *Hericium erinaceus* polysaccharide was analyzed by ion chromatography [30].

The polysaccharide from *Hericium erinaceus* (5 mg) was hydrolyzed separately with 2.5 M CF_3_COOH (3 mL) for 2 h at 121 °C in a sealed glass tube. Then it was air dried with N_2_, and the air-dried sample was washed with methanol and air-dried with N_2_, and the cycle was 3 times. The resulting dry sample was dissolved in 1 mL of distilled water and diluted 100 times after pH was adjusted to neutral. Finally, the samples were evaluated using a HPAEC-PAD instrument with a CarboPac PA20 column (3 mm × 150 mm, 5 μm, Thermo Scientific, Rochester, NY, USA). The mobile phase A, B, D was water, 1 M CH3COONa, and 250 mM NaOH, respectively. The running program was set to 0–13 min, the mobile phase was 2% D; for 13–13.1 min, the mobile phase was 5% B and 2% D; 13.1–20 min, the mobile phase was 20% B and 2% D; 20–21 min, the mobile phase was 80% D; For 21–30 min, the mobile phase was 80% D. The injection volume was 10 μL, the flow rate was 0.5 mL/min, and the temperature of column was 30 °C.

### 2.6. IR Spectrum

The chemical bond and functional groups of *Hericium erinaceus* polysaccharide and its derivatives were analyzed using Fourier transform infrared spectrometer. KBr was used as the background during the measurement.

The powder mixture of 2 mg dry *Hericium erinaceus* polysaccharide sample and 200 mg KBr was ground and pressed into 1 mm thick sheets. The wavelength was set to be in the range of 4000–600 cm^−1^ for infrared spectrum scanning to analyze the primary structure of *Hericium erinaceus* polysaccharide [31].

### 2.7. Determination of Antioxidant Activity In Vitro

#### 2.7.1. Scavenging Ability of DPPH [32]

The VC at the same concentration was used as a positive reference to calculate the mean value. An amount of 2 mL of HEP-W solution and HEP-A solution with a concentration of 1~3 mg/mL was added to different test tubes. Then, 1 mL of DPPH·-ethanol solution at a concentration of 0.1 mmol/L was added to each tube sequentially. The mixture was mixed well and reacted for 30 min at room temperature in the dark. The absorbance value of the solution to be measured at 517 nm was determined, and the above experiment was repeated three times. The scavenging rate was calculated as follows:

E = [1 − (A1 − A2)]/A0 × 100%

#### 2.7.2. ABTS^+^ Radical Scavenging Capacity Determination [33]

The VC at the same concentration was used as a positive reference to calculate the mean value.

First, 0.0384 g ABTS^+^ and 0.0134 g potassium persulfate were dissolved well with deionized water, and the volume was set in a 10 mL volumetric flask, mixed according to the ratio of 1:1, stored in the dark for 12 h, ABTS^+^ solution was prepared, and reserved.

When the measurement was made, it was diluted with deionized water, and the dilution was completed when the absorbance of the diluted solution was 0.70 ± 0.02 at 734 nm, and the value was set to V0. Next, 2 mL of ABTS^+^ diluent was added to different test tubes. Then, 1 mL of HEP-W solution and HEP-A solution was added with a concentration of 1~3 mg/mL. The mixture was mixed well and reacted for 30 min at room temperature in the dark. The absorbance value of the solution to be measured at 734 nm was determined (A1), and the above experiment was repeated three times. The scavenging rate was calculated as follows:

E = (A0 − A1)/A0 × 100%

#### 2.7.3. Determination of Hydroxyl Radical Scavenging Ability [34]

The VC at the same concentration was used as a positive reference to calculate the mean value.

First, to 9 mmol/L FeSO_2_ solution, 9 mmol/L salicylic acid solution, and 1~3 mg/mL HEP-W, HEP-A lion’s Mane polysaccharide solution 1 mL each, 1 mL of 8.8 mmol/L H_2_O_2_ solution was added, mixed well, heated in a 37 °C water bath for 30 min, centrifuged at 25 °C, 8000 rpm for 6 min, and the supernatant was taken at 510 nm to determine A1; Determination of A2 was performed with 1 mL of deionized water instead of H2O2 solution; A0 was determined with 1 mL HEP-W and HEP-A solution as a blank control.

The scavenging rate was calculated as follows:

E = [1 − (A1 − A2)]/A0 × 100%

### 2.8. Effects of Hericium erinaceus Polysaccharides on Intestinal Microbial Metabolites

#### 2.8.1. Preparation of Digestion and Fermentation

Digestion in vitro

The digestion was conducted according to the published reports with minor modifications [35,36].

Phosphate buffer (PBS) was prepared in a volumetric flask of 1.3244 g Na_2_HPO_4_·H_2_O, 2.7879 g Na_2_HPO_4_·7H_2_O and 0.585 g NaCl to 1 L, and the pH was adjusted to 6.9 ± 0.1 for backup. Human saliva α amylase was dissolved in 1 mmol/L of CaCl_2_ to prepare 28.4 U/mL human salivary α amylase, along with 6 mol/L HCl, 52 U/mL pepsin diluted with 1 mg/mL HCl, 6 mol/L NaOH, and pancreatic enzymes.

Fermentation in vitro.

Trace element solutions were prepared according to the recipe requirements in Table S1.

Carbonate–phosphate buffer was prepared as required in Table S2.

The prepared carbonate–phosphate buffer was sterilized at 121 °C for 30 min, cooled to room temperature, filtered with a 0.22 μm filter, and 0.25 g/L of cysteine-hydrochloride was added. The buffer was then transferred to an anaerobic station and the culture continued until the color of the carbonate–phosphate buffer was reduced to colorless, indicating that the liquid was anaerobic for subsequent experiments.

Fresh human fecal samples were collected from five adult volunteers (all of them, aged from 20 to 25, had a healthy diet and no intestinal disease) who had not taken antibiotics within 3 months [37]. (All subjects gave their informed consent for inclusion before they participated in the study. The study was conducted in accordance with the Declaration of Helsinki, and the work was approved by the Ethics Committee of Shanghai Jiao Tong University with approval #B2021153I.)

Each volunteer provided 8 g of fresh fecal samples in a sterile sealed tube which was quickly transferred to an anaerobic workstation, mixed well on a shaker in a 10:1 ratio of carbonate–phosphate buffer and feces, and filtered with 4 layers of sterile gauze twice to obtain the final human fecal inoculum, which was now prepared for immediate use.

Simulated gastrointestinal digestion in vitro

First, 3 g each HEP-W and HEP-A were dissolved in 21 mL PBS and then incubated in a water bath at 37 °C and 150 rpm for 15 min to equilibrate the temperature, after adding 0.5 mL 28.4 U/mL human saliva α amylase to a well-balanced tube, and continuing to shake at 37 °C and 150 rpm for 15 min in a thermostatic shaker. The pH of the inactivated solution was adjusted to 7.0 ± 0.1 after boiling in water to inactivate enzymes. Next, 0.5 mL of pancreatic enzyme was added, 37 °C, 150 rpm, shaken for 90 min, and put in boiling water at 100 °C. Then, the inactivated enzyme was allowed to cool to room temperature, put into a 3500 Da dialysis bag, dialyzed for 24 h, changing the water every 8 h. Freeze-dried to obtain hydro *Hericium erinaceus* polysaccharide HEP-WX and alkali extract *Hericium erinaceus* polysaccharide HEP-AX after digestion of saliva–gastric–intestinal fluid.

#### 2.8.2. In Vitro Fermentation Assay of HEP-W and HEP-A

First, 0.44 g of digested HEP-W and HEP-A were added to each anaerobic tube, with inulin as the positive control group and nothing as the blank control group. (Inulin is widely recognized as the safest and most effective prebiotic in international research. It is utilized by almost all types of beneficial bacteria such as *Lactobacillus* and *Bifidobacterium*, and can even be metabolized by some neutral bacteria like *Anaplasma* and *Clostridium*. Inulin has a significant impact on promoting the growth of beneficial bacteria.) Next, cheesecloth, beaker, anaerobic tube, stopper, pipette tip, aluminum seal, etc. were put into an autoclave and sterilized at 121 °C for 30 min, and the anaerobic tube containing *Hericium erinaceus* samples was put into the anaerobic workstation the day before the experiment. Anaerobic signs, pipettes, scales, graduated cylinders, mini shakers, etc. were put into the anaerobic workstation and wiped and disinfected with alcohol.

The experiment was started when the buffer became colorless, the entire experimental temperature was maintained at 37 °C, and the entire experiment was performed in an anaerobic workstation. An amount of 5 mL of colorless transparent carbonate–phosphate buffer solution was added after anaerobic reduction to the tube containing the experimental polysaccharide sample, and the anaerobic tube containing the colorless buffer and the polysaccharide sample was placed on a vortex mixer for 5 min, and the experimental polysaccharide sample was fully dissolved in the colorless buffer. After that, 1 mL of fecal microbial suspension with the above configuration was added to an anaerobic tube containing the polysaccharide sample and buffer, mixed on a vortex mixer for 3 min, sealed with a rubber cap, and then sealed with an aluminum seal, all of which were performed within 2 h of the volunteer’s feces being removed [38].

#### 2.8.3. Determination of Gas Production

Using a 20 mL sterilized glass syringe, a sterile disposable needle was inserted into the rubber plug of the anaerobic tube, and left until the gas pressure in the tube pushed the glass syringe upward until the glass syringe was stable and no longer moved. During this process, the values displayed by the glass syringe were recorded to record the changes in gas production [39,40].

Samples were taken at 0, 3, 6, 12, 24, and 48 h of simulated fermentation in vitro, and the average value was recorded as gas production at this time.

#### 2.8.4. Determination of pH

The pH value of fermentation liquid was recorded using a pH meter, and samples were taken at 0, 3, 6, 12, 24 and, 48 h of fermentation. The average value was recorded as the pH value of fermentation liquid at this time.

#### 2.8.5. Determination of Short Chain Fatty Acid Content

The fermentation product at 0, 3, 6, 12, 24, and 48 h was centrifuged at 4000 g for 15 min to take 1 mL of supernatant, and then mixed with 0.5 mL of 0.05 mmol/L phosphoric acid, which contained 0.05 mmol/L 4-methylvaleric acid and 8% copper sulfate, and stored in the refrigerator at −20 °C. The contents of SCFAs were calculated according to the calibration curves of respective standards of SCFAs [41,42].

Two microliters of solution to be tested were loaded in GC (Agilent Technologies 6890 N) with a DB-FFAP column (60 m × 0.25 mm × 0.5 μm, Agilent, Santa Clara, CA, USA). Helium gas (He) was used as the carrier gas at a flow rate of 0.7 mL/min. The initial column temperature was kept at 90 °C for 3 min, which was then raised to 130 °C at 15 °C /min and maintained at that temperature for 3 min. After that, the temperature was further increased to 180 °C at 5 °C /min and maintained for 3 min at that temperature.

#### 2.8.6. Changes in Microbiota Composition

DNA extraction and pyrosequencing:

Sequencing bacteria 16s rRNA was used to study the effect of HEP-W and HEP-A on the gut microbiota.

In this study, we extracted DNA from samples that were fermented for 48 h using the Micro DNA Extraction Kit from Omega Reagent Company, Cheyenne, WY, USA. To amplify the variable regions of microorganism V3–V4, we used total DNA and 338F_806R as templates and primers, respectively. The size and quantity of the amplicon library were determined using the Illumina MiSeq Library Quantification Kit and a paired-end library was constructed using standard Illumina sequencing primers. Bidirectional sequencing was performed on the PE300 MiSeq platform to obtain high-quality sequencing data.

Microbial diversity QIIME2 was used to screen the data, the experimental samples were analyzed using α-diversity analysis and β-diversity analysis to analyze the community diversity, and the community composition after 48 h of fermentation was analyzed using colony composition diagram.

## 3. Results and Discussion

### 3.1. Determination Results of Chemical Components of Two Kinds of Polysaccharides

In this experiment, two extraction methods, water extraction and alcohol precipitation and alkali extraction and alcohol precipitation, were used to obtain HEP-W and HEP-A through deproteinization, dialysis, and freeze-drying. The extraction rates were, respectively, 0.3% and 3.1%.

The total sugar, reducing sugar, and uronic acid content of the HEP-W and HEP-A samples are shown in Table 1. The purity of the polysaccharides obtained by both methods was higher and contained fewer impurities, and this result was similar to the results of the purity of the polysaccharides obtained from *Hericium erinaceus* extracted with hot water and alkaline solution obtained by Ding [43].

### 3.2. Determination Results of the Molecular Weight of the Two Polysaccharides

The molecular weight distribution of the two polysaccharides HEP-W and HEP-A was determined by high performance gel chromatography, and the molecular weight distribution of the obtained samples is shown in the following Table 2, where Mw is the weight average molecular weight. The two polysaccharides of *Hericium erinaceus*, HEP-W and HEP-A, consisted of two polysaccharides with different molecular weights, among which the polysaccharides with large molecular weight were the main ones. The results were different from those of Zhang [44] for the heteropolysaccharides HEPF1 and HEPF3 obtained from the *Hericium erinaceus* with different extraction methods, which may cause degradation of some of the polysaccharides, resulting in them having lower molecular weights.

### 3.3. Determination Results of Two Kinds of Polysaccharide Monosaccharide Composition

**Figure 1:**
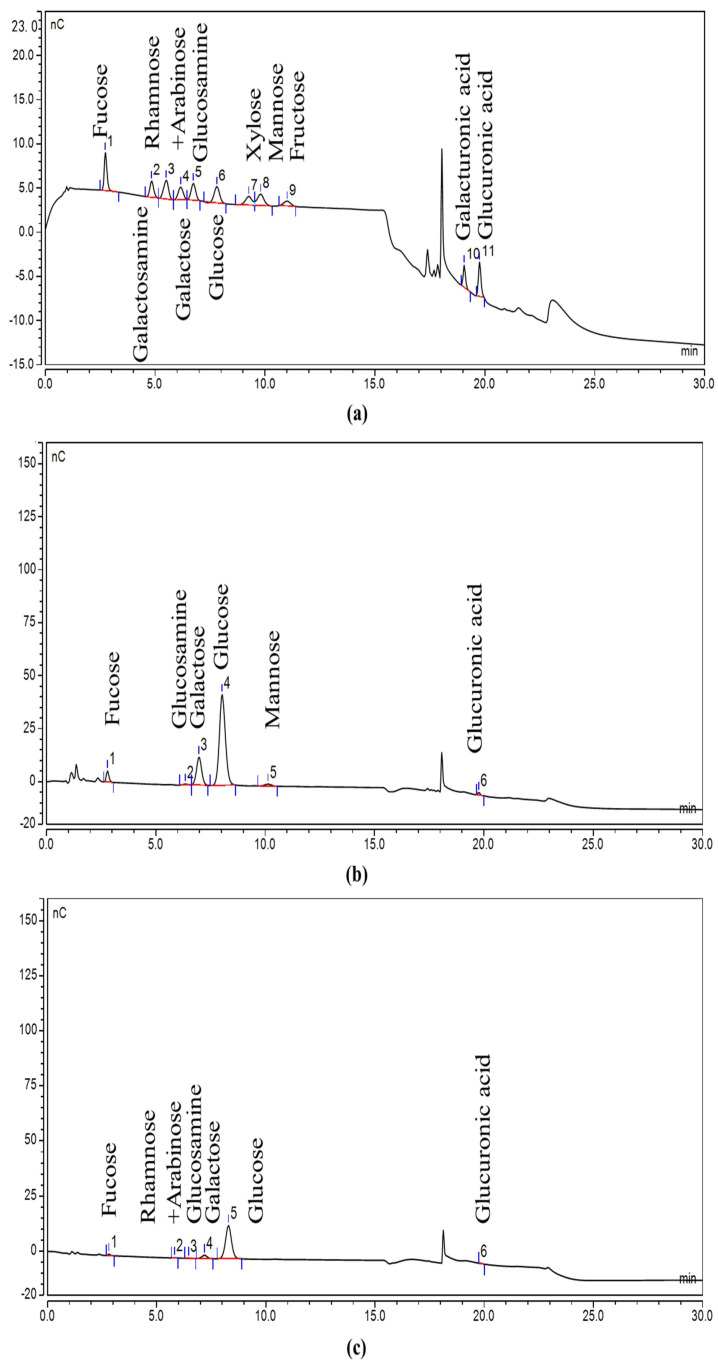
Ion chromatogram: (**a**) mixed standard; (**b**) HEP-W; and (**c**) HEP-A.

The monosaccharide composition of two polysaccharides of *Hericium erinaceus* HEP-W and HEP-A was analyzed, with fucose, galactosamine, rhamnose + arabinose, glucosamine, galactose, glucose, xylose, mannose, fructose, galacturonic acid, and glucuronic acid prepared in a mixed standard solution, and the measured ion chromatograms are shown in Figure 1a. It can be seen from Figure 1b,c that the water-extracted *Hericium erinaceus* polysaccharide HEP-W is composed of fucose, glucosamine, galactose, glucose, mannose, and glucuronic acid, and the alkali-extracted *Hericium erinaceus* polysaccharide HEP-A is composed of fucose, rhamnose + arabinose, glucosamine, galactose, glucose, and glucuronic acid, which shows that HEP-W and HEP-A are heteropolysaccharides mainly composed of glucose. As shown in Table 3, the use of different extraction methods resulted in variations in the monosaccharide composition of HEP-W and HEP-A. The crude alkaline-extracted polysaccharide had a significantly higher total sugar content compared to the water-extracted polysaccharide. Additionally, the decrease in glucose content in HEP-W suggested that some amyloid glucan chains were broken down into oligomeric fragments, which were subsequently removed during alcoholic precipitation.

The results differed from those of HEP-1 extracted by Jia et al. [45]. The reason may be due to the different sources and extraction methods of *Hericium erinaceus*. Hot water extraction can better preserve the molecular structure, but the efficiency is low, and may degrade polysaccharides. The principle of alkaline extraction is that under certain conditions of temperature, time, and pH, the cells swell and destroy the cell wall, from which polysaccharides are released, which is favorable for an increase in the extraction rate.

### 3.4. FTIR Analysis

**Figure 2:**
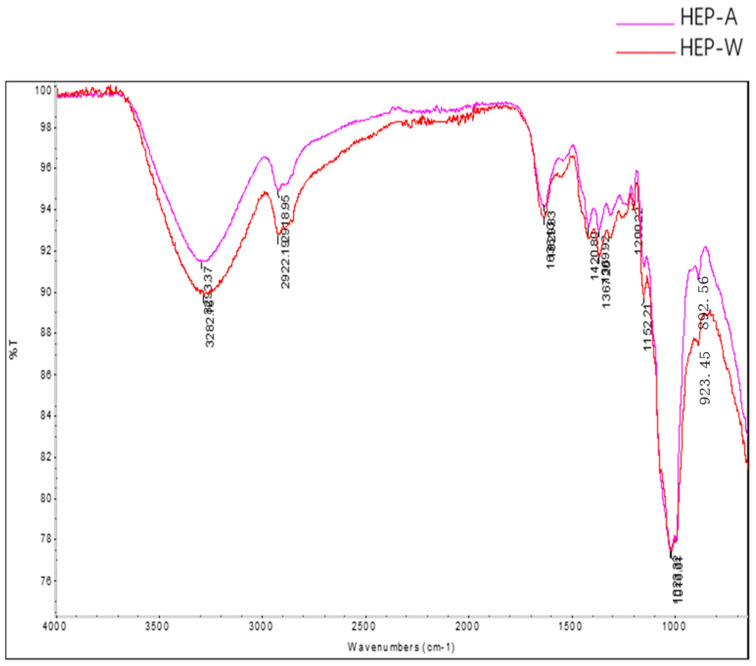
Infrared spectra of HEP-W and HEP-A.

It can be seen from Figure 2 that the characteristic functional groups of HEP-W and HEP-A are similar, and the strong absorption peak at 3293.37 cm^−1^ was the O-H stretching vibration absorption peak of sugar ring. The absorption peak at 2918.95 cm^−1^ was the C-H stretching vibration, and 1629.83 cm^−1^ represented the bending vibration peak of O-H. Further, 1420.80 cm^−1^ and 1369.92 cm^−1^ represented the variable angle vibration of C-H. According to the absorption peaks of 1023.32 cm^−1^ and 1200.22 cm^−1^, it could be determined that the sample contained a pyran ring. The absorption peaks of the β-glycosidic linkage were 892.56 cm^−1^ and 923.45 cm^−1^. The analysis result of the IR spectra suggested that HEP-W and HEP-A were the pyran ring polysaccharide containing β-glycosidic linkage in their structure. This result is consistent with the findings of Ding [43] and Zhang [25].

### 3.5. Antioxidant Activity

In the in vitro antioxidant activity test, VC was used as the positive control substance.

**Figure 3:**
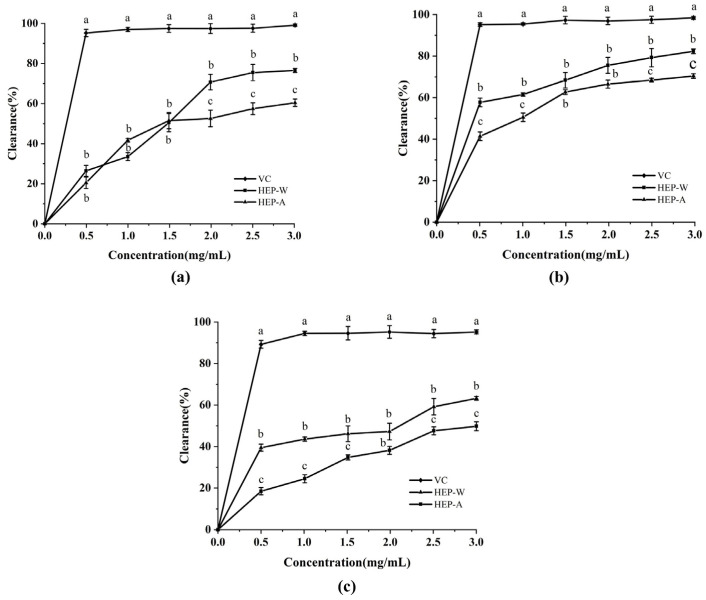
Scavenging ability of HEP-W and HEP-A on free radicals: (**a**) DPPH· radical-scavenging activity; (**b**) ABTS^+^ radical-scavenging activity; and (**c**) hydroxyl radical-scavenging activity. The data are expressed as the mean ± standard deviation. Different letters indicate a significant difference (*p* < 0.05).

The scavenging ability of HEP-W and HEP-A on DPPH· can be seen from Figure 3. ABTS^+^ and hydroxyl radicals were positively correlated with the dose in the range of 0–3 mg/mL, but the scavenging rate was not as high as that of VC. HEP-A had a stronger scavenging ability than HEP-W on DPPH· and ABTS^+^, while its scavenging ability on hydroxyl radicals was weaker than that of HEP-W. 

When the concentration of polysaccharides was 3 mg/mL, the scavenging ability of HEP-W and HEP-A on the DPPH· free radical was 59.9% and 76.3%, respectively. This may be because the magnitude of the antioxidant capacity mainly depends on the molecular weight, chain conformation, and structure of the polysaccharide [46,47]. Because alkali treatment extracts and degrades the macromolecular polysaccharides of *Hericium erinaceus*, so that its solubility increases, more active groups are exposed, and DPPH· is easily captured. This is consistent with the results of the study on the DPPH·-scavenging ability of jujube alkali extraction and polysaccharide extraction by water [48]. When the concentration of polysaccharide was 3 mg/mL, the scavenging ability of HEP-W and HEP-A on ABTS^+^ free radicals was 70.9% and 82.2%, respectively; the scavenging ability of the hydroxyl radical was 62.97% and 50.45%, probably because the carboxyl group of glyoxylic acid in HEP-W played a role in providing a hydrogen and electron transfer agent.

### 3.6. Prebiotic Activities

#### 3.6.1. Total Sugar, Reducing Sugar, and Molecular Weight of HEP-W, HEP-A after Simulated Gastrointestinal Digestion

Table 4 shows that there was a significant change in total sugar, reducing sugar, and molecular weight of HEP-W and HEP-A at different times during gastrointestinal digestion in vitro.

It was found that the total and reducing sugars in HEP-W decreased by 10% and 0.1%, respectively, compared with those in HEP-A before in vitro digestion, and the total and reducing sugars in HEP-A decreased by 11% and about 1%, respectively, which could indicate that most of the two types of polysaccharides of *Hericium erinaceus* were not able to be digested in the in vitro digestive environment. After in vitro simulated digestion, two molecular weights were detected for both types of polysaccharides, and the molecular weights were smaller compared with those before in vitro digestion, but the changes were small, which further proved that both types of polysaccharides were not able to be digested by saliva–gastric–small-intestinal fluid, and they were able to enter into the large intestine for fermentation without any problem. The result was similar with many plant polysaccharides, such as fucosylated glycosaminoglycan from sea cucumber and polysaccharides from Fuzhuan brick tea and kiwifruit [49,50,51].

These results indicated that HEP-W and HEP-A were not degraded by the saliva–gastric–intestinal fluid, and could go smoothly into the large intestine for fermentation.

#### 3.6.2. Gas Production Change Chart during Fermentation

**Figure 4:**
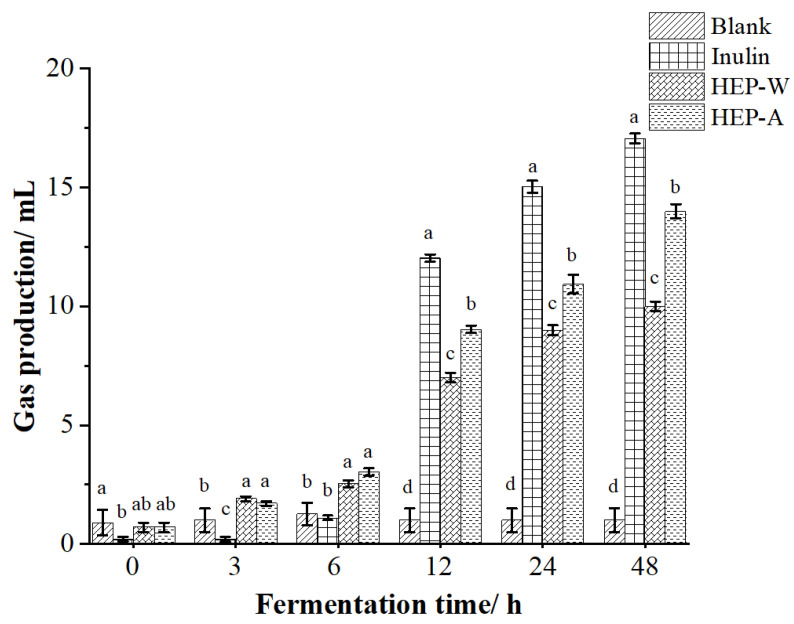
Variation of gas content during fermentation process. The data are expressed as the mean ± standard deviation. Different letters indicate a significant difference (*p* < 0.05).

Intestinal microorganisms produce certain CO_2_, CH_4_, H_2_, and other gases when metabolizing polysaccharides [52]. In the process of in vitro fermentation, the changes in gas production of each group in the experimental group are shown in Figure 4. After 48 h of fermentation, the gas production of the blank control group is the lowest, and the gas production of the experimental group is proportional to the fermentation time, among which the gas production of inulin in the positive control group is the highest. The gas production of HEP-W and HEP-A increased the fastest within 6–12 h, which is speculated to be due to the full metabolic utilization of HEP-W and HEP-A by intestinal microorganisms at 6–12 h, resulting in a faster gas production increase at this time.

The above gas production results proved that both HEP-W and HEP-A could be utilized by intestinal microorganisms to produce gas, and the gas production of HEP-A was higher than that of HEP-W and slightly lower than that of the positive control group.

#### 3.6.3. Diagram of pH Changes during Fermentation

**Figure 5:**
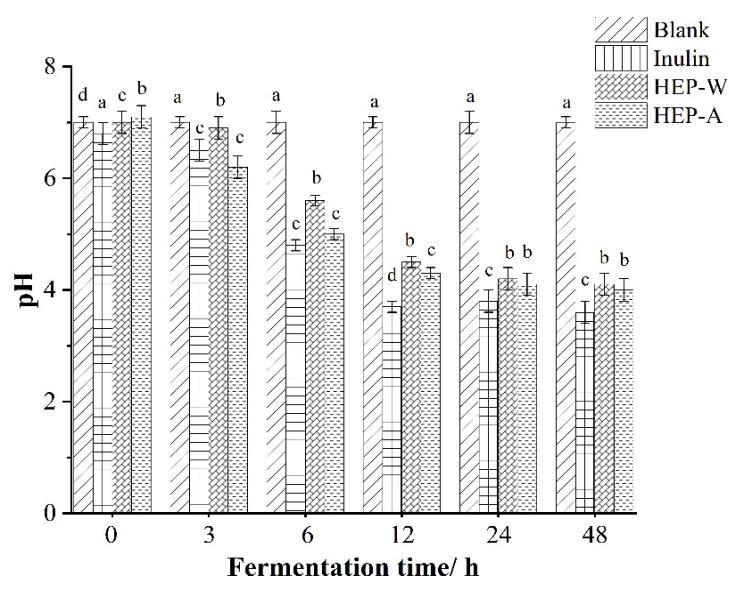
pH value changes during fermentation process. The data are expressed as the mean ± standard deviation. Different letters indicate a significant difference (*p* < 0.05).

The pH value of each component in the fermentation process is shown in Figure 5. After 48 h of fermentation, the pH value of the blank control group had no significant change, while the pH value of HEP-W and HEP-A group and inulin in the positive control group was inversely proportional to the fermentation time, and significantly decreased compared with 0 h. The pH value of inulin in the positive control group decreased the most. In the polysaccharide group, the pH of HEP-A decreased the most, followed by the HEP-W group. The results showed that HEP-W and HEP-A could regulate the metabolism of intestinal microorganisms to a certain extent. At 3–12 h, the pH values of HEP-W and HEP-A in the positive control group and the polysaccharide group declined the fastest, which may be because at 3–12 h, intestinal microorganisms utilized the two polysaccharides at the fastest rate in the metabolic process, and produced organic acids and short-chain fatty acids by fully metabolizing polysaccharides. Thus, the pH value drops the fastest at this time [53]. At 12–48 h, pH values of HEP-W group and HEP-A group did not change significantly, which may be attributed to the decrease in the polysaccharide content of two kinds of *Hericium erinaceus* in the anaerobic tube, less polysaccharide available for intestinal microorganisms, and the consumption of organic acids in pre-metabolism as energy. Therefore, it can be seen from the above pH value results that both HEP-W and HEP-A can reduce the pH value and promote the growth of beneficial bacteria, among which HEP-A has the best effect, followed by HEP-W.

#### 3.6.4. Changes in Short-Chain Fatty Acid Content during Fermentation

**Figure 6:**
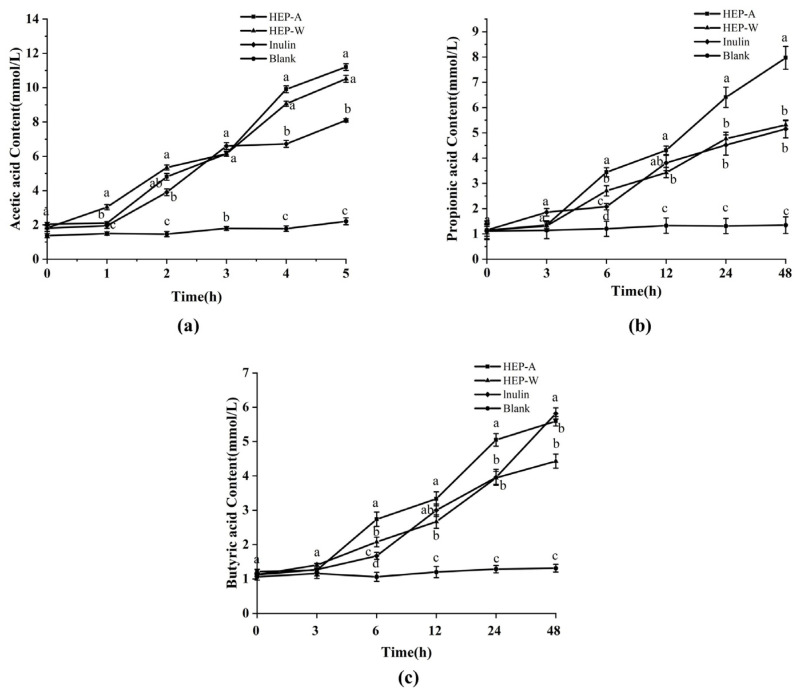
Short-chain fatty acid changes during fermentation: (**a**) concentrations of acetic acid; (**b**) concentrations of propionic acid; and (**c**) concentrations of butyric acid. The data are expressed as the mean ± standard deviation. Different letters indicate a significant difference (*p* < 0.05).

It can be seen from Figure 6a that, compared with the blank group, intestinal microorganisms can use HEP-W and HEP-A to produce short-chain fatty acids, and the content of short-chain fatty acids is proportional to the time of in vitro simulated fermentation, and during the fermentation process acetic acid production in medium > propionic acid production > butyric acid production. It can be seen from Figure 6a that in the experimental group, the content of acetic acid in the HEP-W group and HEP-A increased significantly, indicating that HEP-W and HEP-A are in the intestinal tract. Under the influence of microorganisms in the intestinal tract, more acetic acid is metabolized.

It can be seen from Figure 6b that the content of propionic acid in the experimental group was lower than that of acetic acid, and the HEP-A group had the largest increase. With the increase in fermentation time, HEP-A changed rapidly after 12 h, and its content was 8.01 mmol/L at 48 h, exceeding the 5.17 mmol/L of the positive control group, and the propionic acid content of HEP-W group was 5.31 mmol/L, slightly higher than the positive control group. This shows that the addition of HEP-W and HEP-A promotes the metabolism of intestinal microorganisms to produce propionic acid. 

The butyric acid content in the experimental group (Figure 6c) showed an overall upward trend during the fermentation process. At 48 h, the butyric acid content of the HEP-A group was 5.64 mmol/L, slightly lower than that of the positive control group (5.79 mmol/L), and higher than that of the HEP-W group.

To sum up, HEP-W and HEP-A produce short-chain fatty acids, mainly acetic acid, followed by propionic acid and butyric acid. The addition of HEP-W and HEP-A can promote the production of acetic acid, propionic acid, and butyric acid. The content of acetic acid in HEP-W is greater than that in HEP-A, and the content of propionic acid and butyric acid in HEP-A is greater than that in HEP-W.

It is speculated that the reason is that the polysaccharides of *Hericium erinaceus* obtained by different extraction methods contain various glycosidic bond conformations such as β→1,3; β→1,6; β→1,4, etc., and the intestinal microorganisms that metabolize HEP-W and HEP-A are different, and these different intestinal microorganisms produce short-chain fatty acids through different enzymatic pathways, mainly divided into direct butyric acid production and indirect butyric acid production using acetic acid and lactic acid, thus producing different results on short-chain fatty acids.

### 3.7. Changes in Microbiota Composition

#### 3.7.1. Alpha Diversity Index

It can be seen from Table 5 that the average coverage rate of the experimental group after 48 h of fermentation is 100%, indicating that the coverage rate of the experimental sequence is high, which may reflect the authenticity of the microbial community in this experiment. The Chao-1 and ACE indices can estimate the community richness of the sample. The Shannon and Simpson index can be used to evaluate the community diversity of the sample.

The Chao and ACE indices of HEP-W were found to be higher than those of HEP-A, and both were higher than those of the blank control group. This suggests that the inclusion of two polysaccharides from *Hericium erinaceus* can enhance the diversity of the intestinal microbial community. The Shannon index of the two kinds of *Hericium erinaceus* polysaccharides in the experimental group was 3.81, both of which were greater than those of the blank group, and the Shannon index was directly proportional to the diversity of the colony. The lower the Simpson index, the higher the species diversity, and the Simpson index of the HEP-W group in the experimental group was lower than that of the positive control group inulin. 

The above results indicated that the addition of two polysaccharides from *Hericium erinaceus* could increase the diversity and richness of the microbial community.

#### 3.7.2. Colony Composition Analysis at the Phylum Level and Family Level

**Figure 7:**
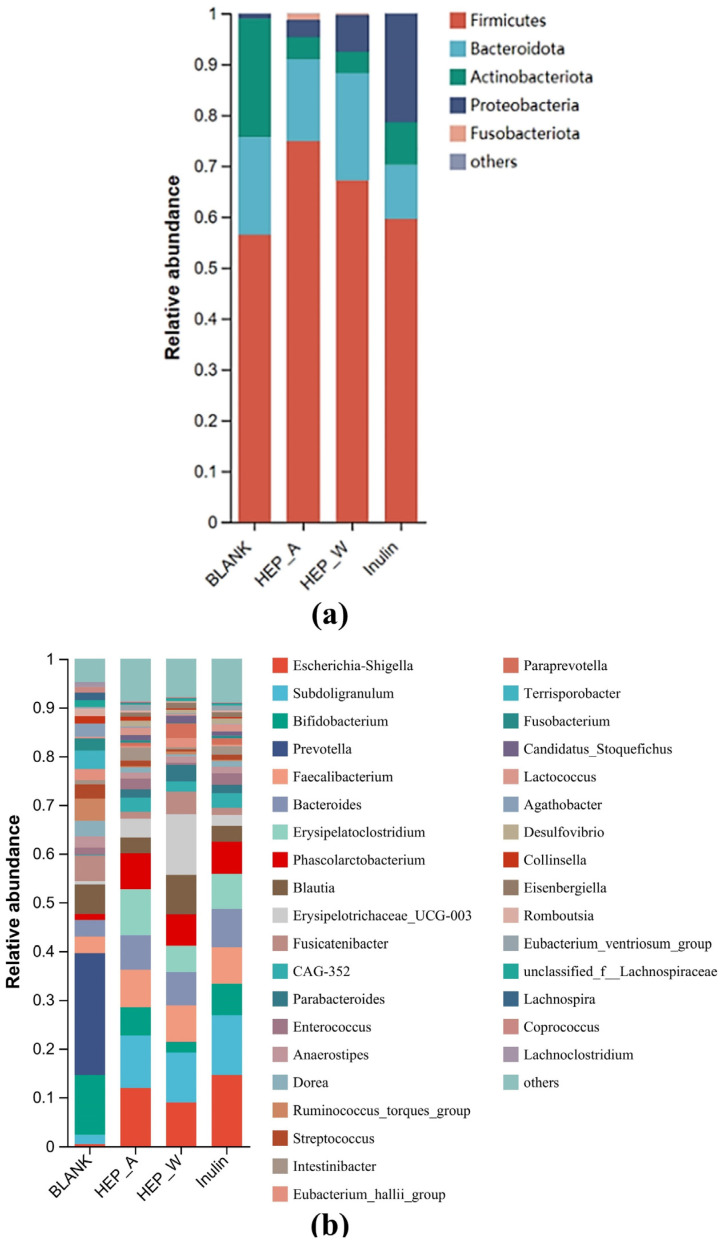
Colony composition diagram: (**a**) phylum level; and (**b**) genus level.

As shown in Figure 7a, experimental groups were mainly composed of Firmicutes, Bacteroidetes, Actinobacteria, and Proteobacteria at the phylum level. After 48 h of fermentation, the relative abundance of Bacteroidetes in the HEP-W and HEP-A groups were significantly higher than that of the blank control group. Among them, Firmicutes of the HEP-A group was higher than that of the HEP-W group, while Bacteroidetes was lower than that of HEP-W group. *Fusobactierium*, *Eubacterium*, *Clostridium*, and *Roseburia* in the Firmicutes can directly degrade β-glucan into glucose, which undergoes glycolysis and other pathways to produce butyric acid. Bacteroidetes can degrade polysaccharides into gluco-oligosaccharides or oligosaccharides, which can then be used by *bifidobacteria* and *lactobacilli* in the intestinal flora to produce lactic acid, and finally butyric acid is produced by lactic-acid-using bacteria, which explains the results of short-chain fatty acids with high levels of butyric acid produced in the HEP-A group and high levels of acetic acid in the HEP-W group.

At the level of genus (Figure 7b), *Faecalibacterium*, *Bacteroides*, *Parabacteroides*, *Lactococcus*, and *Phascolarctobacterium* in the HEP-W and HEP-A groups increased significantly compared with the control group after fermentation for 48 h. These genera are known acidogenic dominant genera, capable of producing short-chain fatty acids in the process of metabolism.

Compared with the blank control group (Blank), the relative abundance of *Prevotella* in the HEP-W and HEP-A groups decreased significantly. *Prevotella* spp. are anaerobic Gram-negative bacteria, and it has been found that *Prevotella* spp. may cause local and systemic diseases in humans, including hypertension [54], periodontitis [55], intestinal dysbiosis [56], rheumatoid arthritis [57], metabolic disorder [58], etc.

These results showed that HEP-W and HEP-A had a potential prebiotic effect by improving the relative abundances of some beneficial gut microbiota. Thus, the two polysaccharides are utilized in different ways to produce beneficial metabolites and regulate intestinal health.

#### 3.7.3. Community Heatmap Analysis

**Figure 8:**
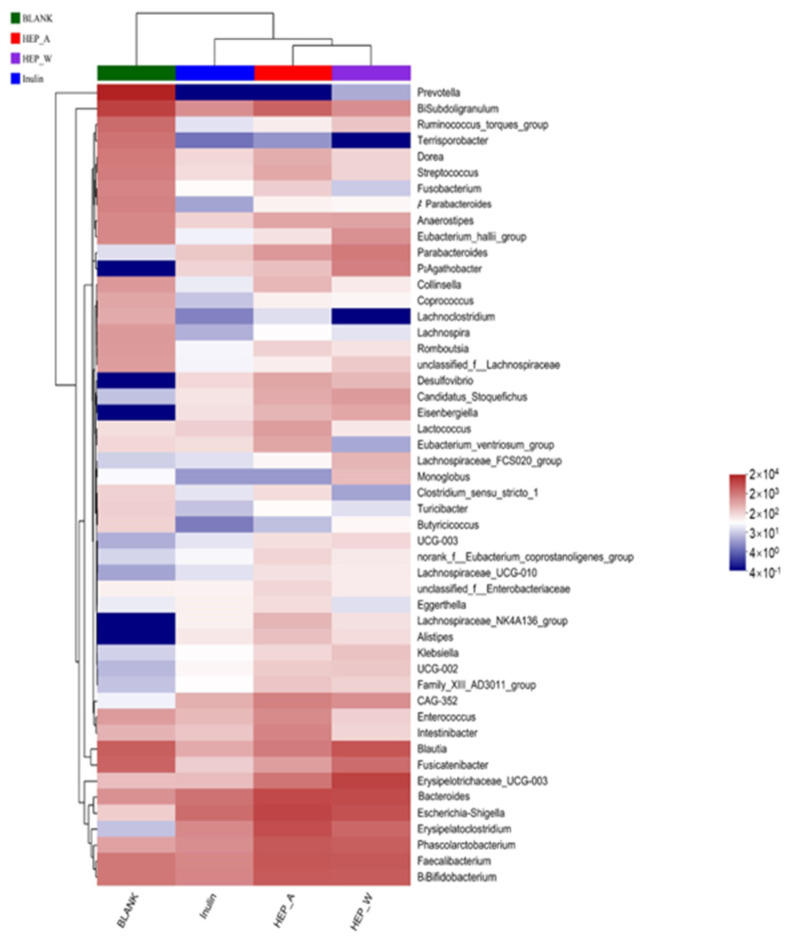
Community Heatmap (Genus level).

The colony heat map (Figure 8) represents the size of the data by color and presents the information of the colony composition, which further proves that the addition of two *Hericium erinaceus* polysaccharides can increase the relative abundance of beneficial bacteria such as *Faecalibacterium* and Bacteroides. The relative abundance of the genus *Agathobacter* was higher than that of the blank control group. The genus *Agathobacter* is an anaerobic Gram-positive bacterium and a new species of *Lachnospiraceae*. Studies have shown that it can increase short-chain fatty acids, especially butyl, to alleviate chronic constipation [59].

It can also be found through the heat map that the relative abundance of *Blautia* increased in the HEP-W group, and *Blautia* is also a species of *Lachnospiraceae*, which is the dominant intestinal microorganism. Some studies have found that *Blautia* can produce acetic acid, activate GPR41 and GPR43, and alleviate obesity by inhibiting the insulin signaling pathway and fat accumulation in adipocytes [60], and some researchers have found that *Blautia* is a common and exclusive anaerobic bacteria, which can maintain the balance of the intestinal environment and prevent inflammation by up-regulating intestinal regulatory T cells and producing SCFAs [61].

The colony heat map further showed that the addition of two polysaccharides from *Hericium erinaceus* can promote the proliferation of beneficial bacteria, increase the content of short-chain fatty acids, and maintain intestinal stability.

#### 3.7.4. Circos Diagram

The Circos diagram describes the visual circle of the corresponding relationship between the experimental group and the colony, which reflects the proportion of the dominant species in each experimental group and the distribution of each dominant species in each experimental group.

**Figure 9:**
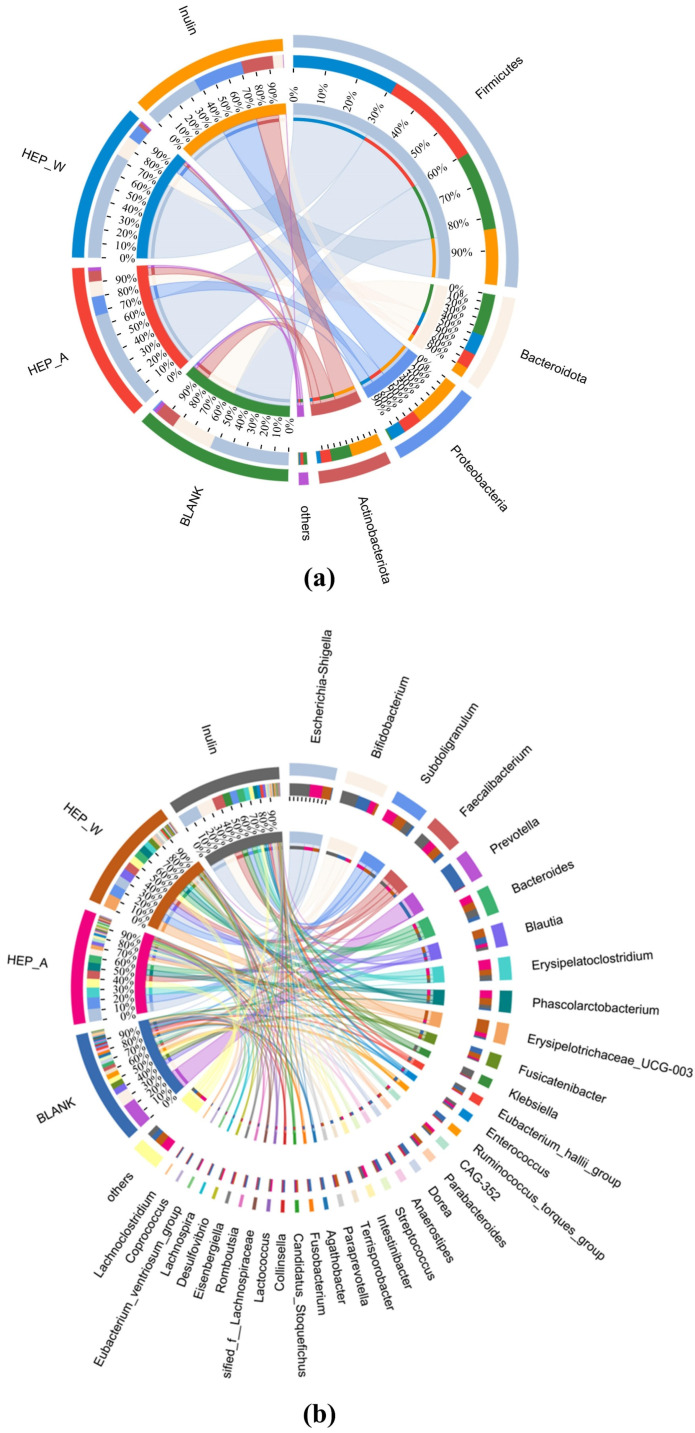
Circos: (**a**) phylum level; and (**b**) genus level.

According to the phylum-level Circos diagram in Figure 9a, it can be seen that there are four dominant bacterial phyla in the experimental group, namely Firmicutes, Bacteroidetes, Actinobacteria, and Proteobacteria. From the Circos diagram at the genus level in Figure 9b, it can be found that the main genera in the experiment are *Bacteroides*, *Subdoligranulum*, *Bifidobacterium*, *Escherichia-Shigella*, and *Faecalibacterium*. It can be seen from the figure that in Blank, HEP-W, HEP-A, and inulin, the proportions of *Bacteroidetes* were 5.3%, 29%, 31%, and 35%, respectively; the proportions of *Parabacteroides* were 13%, 29%, 30%, and 29%, respectively; and the proportions of *Koalabacter* were 5.7%, 30%, 34%, and 30%. These three genera are all well-known beneficial bacteria, which can metabolize short-chain fatty acids and maintain intestinal stability [62].

The Circos diagram shows that the addition of HEP-W and HEP-A can promote the change in the colony composition of intestinal microorganisms, and the addition of two polysaccharides of *Hericium erinaceus*, HEP-W and HEP-A, can promote the increase in the relative abundance of beneficial bacteria and have a prebiotic effect. However, the differences in extraction methods cause differences in the structure and molecular weight of HEP-W and HEP-A, resulting in differences in the colonization of intestinal microorganisms.

## 4. Conclusions

In this paper, we investigated the digestion and in vitro fermentation of the two polysaccharides (HEP-W and HEP-A) under simulated gastrointestinal conditions and their effects on the intestinal microbiota by two different extraction methods: aqueous and alkaline extraction and alcoholic precipitation of HEP-W and alkaline extraction of HEP-A. The results showed that HEP-W and HEP-A were substantially degraded and used by human intestinal microbiota during fermentation, which could significantly increase the production of SCFAs. It is noteworthy that because of the different cultivation conditions, species differences, and extraction methods of *Hericium erinaceus*, there may be significant structural differences between HEP-W and HEP-A, which result in different microbial community changes.

The structural study of the two *Hericium erinaceus* polysaccharides in this study is not sufficient, and the study of the molecular mechanism of *Hericium erinaceus* polysaccharides with different solubility, molecular weight, and chemical structure to regulate the production of butyric acid by intestinal flora will surely bring positive and useful additions to the field of target regulation of intestinal flora.

## Figures and Tables

**Table 1:** Results of determination of the chemical composition of two polysaccharides.

Samples	Total Sugar (%)	Reducing Sugar (%)	Uronic Acid (%)
HEP-W	76.69 ± 0.13 ^b^	2.91 ± 0.08 ^e^	3.68 ± 0.18% ^d^
HEP-A	83.92 ± 0.09 ^a^	5.26 ± 0.15 ^c^	2.83 ± 0.12% ^e^

The data are expressed as the mean ± standard deviation. Different letters indicate a significant difference (*p* < 0.05).

**Table 2:** Molecular weight of polysaccharides.

Samples	Mn	Mw	Mw/Mn	Area (%)
HEP-W	441,819 ^b^	597,979 ^a^	1.35 ^b^	87.66 ^a^
	1111 ^d^	1614 ^d^	1.46 ^a^	12.34 ^d^
HEP-A	423,167 ^b^	589,434 ^a^	1.39 ^b^	60.92 ^b^
	1060 ^cd^	1546 ^c^	1.46 ^a^	39.08 ^c^

The data are expressed as the mean ± standard deviation. Different letters indicate a significant difference (*p* < 0.05).

**Table 3:** Molar percentage of HEP-W and HEP-A monosaccharides (%).

Samples	Fucose	Rhamnose + Arabinose	Glucosamine	Galactose	Glucose	Mannose	Glucuronic Acid
HEP-W	4.00	-	0.56	19.37	73.59	1.71	0.78
HEP-A	1.54	0.26	0.28	7.93	89.59	-	0.38

**Table 4:** Results of total sugar, reducing sugar, and molecular weight of HEP-W and HEP-A after digestion in vitro.

Samples	Total Sugar (%)	Reducing Sugar (%)	Mn	Mw	Mw/Mn
HEP-W	67.97 ± 0.14 ^a^	2.86 ± 0.07 ^d^	386,887 ^c^	462,282 ^b^	1.19 ^b^
			1104 ^f^	1563 ^f^	1.42 ^a^
HEP-A	73.77 ± 0.16 ^b^	4.48 ± 0.11 ^c^	383,951 ^c^	470,080 ^a^	1.22 ^b^
			21,060 ^e^	29,516 ^d^	1.40 ^a^

The data are expressed as the mean ± standard deviation. Different letters indicate a significant difference (*p* < 0.05).

**Table 5:** α diversity index of each experimental group.

Samples	Sobs	ACE	Chao	Shannon	Simpson	Coverage
Inulin	163 ^a^	163 ^a^	163 ^a^	3.83 ^a^	0.045827 ^b^	1
HEP-A	161 ^a^	161 ^a^	161 ^b^	3.81 ^a^	0.045074 ^b^	1
HEP-W	155 ^b^	155 ^c^	155 ^c^	3.81 ^a^	0.042790 ^c^	1
Blank	147 ^c^	147 ^d^	147 ^d^	3.79 ^a^	0.049469 ^a^	1

The data are expressed as the mean ± standard deviation. Different letters indicate a significant difference (*p* < 0.05).

## Data Availability

The datasets generated for this study are available on request to the corresponding author.

## Supplementary Materials

The following supporting information can be downloaded at: https://www.mdpi.com/article/10.3390/polym15204165/s1, Table S1: Trace element solution preparation table; Table S2: Carbonate-phosphate buffer solution preparation table; Figure S1: Informed Consent Statement.
