# Supplementary material for: Antioxidant Activities and Prebiotic Activities of Water-Soluble, Alkali-Soluble Polysaccharides Extracted from the Fruiting Bodies of the Fungus Hericium erinaceus
Source: Polymers (Basel). 2023 Oct 20;15(20):4165. doi: 10.3390/polym15204165 (PMC10611342; doi:10.3390/polym15204165)
Supplement: Supplementary file 1 [file polymers-15-04165-s001.zip › polymers-2578987-supplementary/Informed Consent Statement.pdf]

Informed Consent Statement

Dear _________:

We are inviting you to participate in a research study on the effects of edible mushroom polysaccharides on human intestinal flora. This informed consent form provides you with information about the purpose, process, liability, and privacy issues of this study. You are advised to read the study carefully and ask the study leader or researcher if you do not understand anything or if you have any questions or the researcher so that they can be answered in a timely manner.

Your participation in this study is completely voluntary.

Purpose of the Study: Edible mushrooms are large fungi, i.e., mushrooms that are available for human use and have a long history of use in a wide variety of species. They are diverse and have a long history. The polysaccharide component of edible mushrooms mainly exists in the mycelium and fruiting bodies of edible mushrooms, and is the main active component of edible mushrooms. It is the main active ingredient of edible fungi, which not only provides essential nutrients for microorganisms, but also has the function of regulating the ecological stability of human intestinal flora.

It can not only provide essential nutrients for microorganisms, but also has the function of regulating the ecological stability of human intestinal flora. In recent years, more and more studies have shown that the polysaccharide components in edible mushrooms can regulate the structure and function of intestinal flora to inhibit and prevent diseases. The polysaccharides of edible mushrooms are metabolized in the intestinal after decomposition and metabolism in the intestinal tract, acetate, propionate, butyrate and so on can provide energy for the human body, protect the intestinal epithelial barrier, and prevent diseases, protecting the intestinal epithelial barrier, preventing, and alleviating metabolic syndrome, thus maintaining pH stability in the intestinal tract, and ensuring the health of human body. This can maintain pH stability in the intestinal tract and ensure the health of the human body. In the present study, we used polysaccharide of *Hericium erinaceus*, one of the edible mushrooms, as the fermentation substrate, and used it as the fermentation substrate in the in vitro modeling experiment. In the present

study, we used polysaccharide from *Hericium erinaceus*, one of the edible fungi, as the fermentation substrate, and conducted in vitro modeling fermentation experiments to provide basic data on the utilization mechanism of polysaccharide from *Hericium erinaceus* by intestinal flora, and to provide a basis for further research on the utilization mechanism of polysaccharide from *Hericium erinaceus*. This will provide basic data for the utilization mechanism of polysaccharides in intestinal flora and provide ideas for further research on the application of polysaccharides in products.

Research process: If you are willing to participate in this study, you and other volunteers will be sequentially numbered. You need to ensure that you have not taken any antibiotic drugs, probiotic products, or any intestinal diseases within six months. products and have no intestinal diseases. During the experiment, we will arrange someone to take a stool sample for you. Your stool sample will be used only for this study.

Responsibility: If you agree to participate in this study, you have the following responsibilities: to provide a true picture of your medical history and current physical condition; not to take restricted medications, foods, etc.

Privacy Issues: If you agree to participate in this study, we will ensure that any personal information you provide during your participation will be treated with absolute confidentiality and will not be disclosed to anyone outside of the experimental group.

Informed Consent Signature: I have fully read and have been fully informed of the purpose, content, and risks of this study and risks of this study, and all my questions have been fully answered by the researcher, and I am voluntarily participating in this project.

Signature of subject: _______________

Dates: _______________
